# Delayed Effect of Dendritic Cells Vaccination on Survival in Glioblastoma: A Systematic Review and Meta-Analysis

**DOI:** 10.3390/curroncol29020075

**Published:** 2022-02-04

**Authors:** Salvatore Cozzi, Masoumeh Najafi, Marzieh Gomar, Patrizia Ciammella, Cinzia Iotti, Corrado Iaccarino, Massimo Dominici, Giacomo Pavesi, Chiara Chiavelli, Ali Kazemian, Amin Jahanbakhshi

**Affiliations:** 1 Radiation Therapy Unit, Azienda USL-IRCCS di Reggio Emilia, 42122 Reggio Emilia, Italy; Salvatore.Cozzi@ausl.re.it (S.C.); Patrizia.ciammella@ausl.re.it (P.C.); cinzia.iotti@ausl.re.it (C.I.); 2 Skull Base Research Center, Rasool Akram Hospital, Iran University of Medical Sciences, Tehran 14535, Iran; najafi.mas@iums.ac.ir; 3 Radiation Oncology Research Center, Iran Cancer Institute, Tehran University of Medical Sciences, Tehran 1416753955, Iran; m.gomar1365@gmail.com (M.G.); Kazemian@tums.ac.ir (A.K.); 4 Department of Biomedical, Metabolic and Neural Sciences, University of Modena and Reggio Emilia, 41121 Modena, Italy; corrado.iaccarino@unimore.it (C.I.); giacomo.pavesi@unimore.it (G.P.); 5 Neurosurgery Division, University Hospital of Modena, 41125 Modena, Italy; 6 Department of Medical and Surgical Sciences for Children & Adults, Division of Oncology, University-Hospital of Modena and Reggio Emilia, 41121 Modena, Italy; massimo.dominici@unimore.it; 7 Laboratory of Cellular Therapy, Department of Medical and Surgical Sciences for Children & Adults, Division of Oncology, University of Modena and Reggio Emilia, 41121 Modena, Italy; chiara.chiavelli@unimore.it; 8 Stem Cell and Regenerative Medicine Research Center, Iran University of Medical Sciences, Tehran 14535, Iran

**Keywords:** glioblastoma, immunotherapy, dendritic cell vaccination, checkpoint inhibitor, survival

## Abstract

Background: Dendritic cell vaccination (DCV) strategies, thanks to a complex immune response, may flare tumor regression and improve patients’ long-term survival. This meta-analysis aims to assess the efficacy of DCV for newly diagnosed glioblastoma patients in clinical trials. Methods: The study databases, including PubMed, Web of Knowledge, Google Scholar, Scopus, and Cochrane, were searched by two blinded investigators considering eligible studies based on the following keywords: “glioblastoma multiforme”, “dendritic cell”, “vaccination”, “immunotherapy”, “immune system”, “immune response”, “chemotherapy”, “recurrence”, and “temozolomide”. Among the 157 screened, only 15 articles were eligible for the final analysis. Results: Regimens including DCV showed no effect on 6-month progression-free survival (PFS, HR = 1.385, 95% CI: 0.822–2.335, *p* = 0.673) or on 6-month overall survival (OS, HR = 1.408, 95% CI: 0.882–2.248, *p* = 0.754). In contrast, DCV led to significantly longer 1-year OS (HR = 1.936, 95% CI: 1.396–2.85, *p* = 0.001) and longer 2-year OS (HR = 3.670, 95% CI: 2.291–5.879, *p* = 0.001) versus control groups. Hence, introducing DCV could lead to increased 1 and 2-year survival of patients by 1.9 and 3.6 times, respectively. Conclusion: Antitumor regimens including DCV can effectively improve mid-term survival in patients suffering glioblastoma multiforme (GBM), but its impact emerges only after one year from vaccination. These data indicate the need for more time to achieve an anti-GBM immune response and suggest additional therapeutics, such as checkpoint inhibitors, to empower an earlier DCV action in patients affected by a very poor prognosis.

## 1. Introduction

Glioblastoma multiforme (GBM), the most common primary brain tumor, represents about half of the malignant glioma tumors in adults. The overall incidence of this tumor has been estimated to be 3.2 per hundred thousand populations with a median survival of 15 to 17 months [1]. The globally accepted therapeutic approach for GBM includes surgical resection, radiotherapy, and chemotherapy by temozolomide. However, its recurrence is very frequent, with a five-year survival rate of about 5% considering a maximal surgical resection and adjuvant therapies being achievable [2,3,4]. This progressive and invasive behavior necessitates the development of novel treatments [5].

Tumor cells, especially in brain tumors, can evade the immune cells via different mechanisms, such as antigenic modulation, lowering immunogenicity, and immune suppression [6,7]. Immunotherapy is progressively becoming an effective approach for activating the immune system to recognize and destroy tumor cells [8,9]. The application of immunotherapy for the treatment of different malignant tumors is discussed elsewhere, especially in metastatic settings [10,11,12,13]. However, immunotherapy in glioblastoma is much more challenging compared to other solid tumors because of its infiltrative nature and the complex structure of the blood–brain barrier in various parts of the tumor territory. Some clinical trials have been performed to assess the efficacy and safety of immunotherapy with different regimens. Overall, there are two main immunotherapy approaches: passive immunotherapy (with the aim to activate the immune system by monoclonal antibodies and immune checkpoint modulators to confer antitumor response), and active immunotherapy or vaccination (by presenting tumor antigens that stimulate the immune system to produce an endogenous anti-tumor response, leading to the long-term recognition and destruction of the tumor cells) [14]. In the latter type, viral vectors and dendritic cells (DC) have been applied as stimulators and modulators [15]. DCs act as coordinators of the innate immune response by releasing activating cytokines for cytotoxic lymphocytes and NK cells [16]. They present processed antigens to B and T lymphocyte subsets, leading to activation and memory induction [17].

Using dendritic cell vaccination (DCV) to induce tumor regression and improve patients’ long-term survival has been demonstrated in several solid tumors [18]. Variability in DCV protocols includes different activation treatments, such as peptide, tumor antigen RNA, and whole tumor lysates, as well as combination therapy with immunomodulators [19]. Several studies have evaluated the treatment response to DCV in glioblastoma, but there are considerable inhomogeneities in the results. These variations in the results could be due to various methods in DCV preparations, concomitant treatments, or differences in patients’ disease status. In this study, we analyzed the available clinical trials and focused on the time period in which the effect of DCV can become evident. We argue that a short life expectancy for glioblastoma may mask the effect of DCV. The results give us some explanation for such discrepancies in the outcomes of many clinical trials.

## 2. Materials and Methods

### 2.1. Study Selection

**Figure 1:**
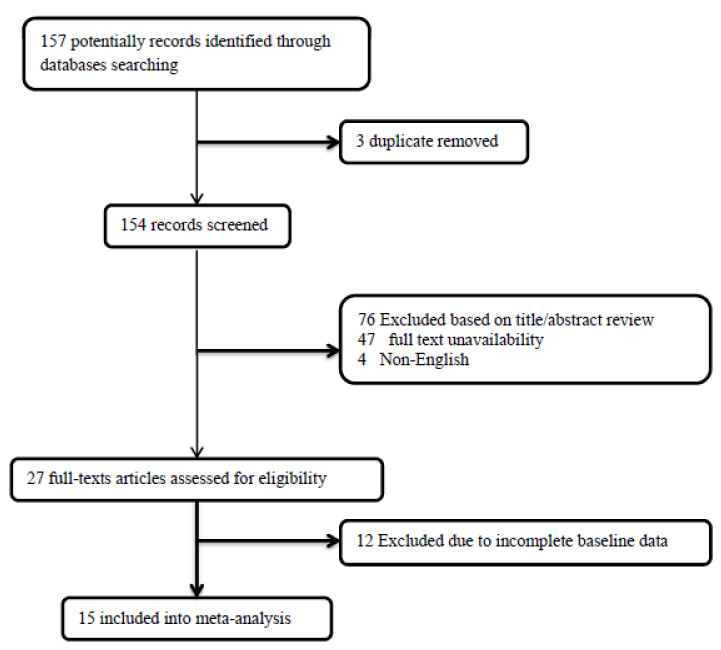
The flowchart of screening the eligible studies.

The present systematic review and meta-analysis followed the guidelines for the Preferred Reporting Items for Systematic Review and Meta-Analysis (PRISMA) revised in 2015 [20] and was generated by the following question: what is the clinical impact of DCV on GBM patient survival? Registration in PROSPERO, by the time of completion of the work, was not a routine local research protocol. Therefore, we do not have a registration number, although our search shows there is no similar registered study in PROSPERO. Databases including Medline, Web of Knowledge, Google Scholar, Scopus, and Cochrane were searched by two blinded investigators for all eligible studies based on the considered keywords, including “glioblastoma multiforme”, “dendritic cell”, “vaccination”, “immunotherapy”, “immune system”, “immune response”, “chemotherapy”, “recurrence”, and “temozolomide”. The inclusion and exclusion criteria were as follows: (1) prospective clinical trials (in different phases I/II/III) evaluating survival in patients suffering from newly diagnosed GBM and scheduling for dendritic cell vaccination with and without temozolomide chemotherapy; (2) studies published in the English language; (3) studies with unclear or irreproducible results (i.e., lack of clear outcomes or presence of errors in methodology and/or analyses) were all excluded; (4) lack of access to the manuscript’s full text was also considered an exclusion criterion, unless the abstracts had enough data for our analysis; (5) case reports, case series, and review papers were all excluded. As shown in the flow diagram of the study selection (Figure 1), 157 articles were initially collected by database searching. After removing 3 articles due to evidence of duplication, 154 records were primarily under-screened. Based on the mentioned criteria, 127 records were excluded, and the remaining 27 citations were assessed for further eligibility. Of those, 12 were also excluded due to the incompleteness of the data and contents. Finally, 15 articles were eligible for the final analysis [21,22,23,24,25,26,27,28,29,30,31,32,33,34,35] (Table 1).

### 2.2. Data Extraction and Quality Assessment

The data collection was independently performed by two unblinded reviewers on structured collection forms. We resolved disagreements by consensus or by involving a third person. The study quality was evaluated based on the following criteria: (1) the systematic review and meta-analysis based on the questions primarily described and formulated; (2) inclusion and exclusion criteria predefined in the studies as eligibility criteria; (3) searching the literature performed on a systematic and comprehensive approach; (4) to minimize the bias, the full texts of the article were dually reviewed; (5) the quality of the included studies was rated independently by the reviewers for appraising internal validity; (6) the studies’ characteristics and findings were comprehensively listed; (7) the publication and risk of bias were listed; and (8) heterogeneity was also assessed. The nine-star Newcastle–Ottawa Scale (NOS) scoring system was employed to assess the methodological quality of all eligible studies. In this quality assessment technique, each study was assessed qualitatively for three criteria: the selection of the study groups, the comparability of the study groups, and the ascertainment of the outcome. The studies awarded 7 stars or more were deemed to be of high quality. Any disagreement was resolved by discussion in the whole study team. The endpoints of this meta-analysis are overall survival, progression-free survival, and toxicity associated with dendritic cell vaccination. Mid-term survival is considered outcomes encountered less than one year after treatment.

### 2.3. Statistical Analyses

The dichotomous variables are reported as proportions and percentages. The pooled likelihood of improving the survival of patients on different regimens was assessed and presented by the hazard ratio (HR) and 95% confidence interval (CI) as summary statistics. The fixed effects or random effects (in the case of significant heterogeneity across the data) models were used to obtained pooled dichotomous data using the mean difference (MD) followed by reporting 95% CIs and its corresponding *p* values. Cochrane’s Q test was used to determine the statistical heterogeneity. This test was complemented with the I2 statistic, which quantifies the proportion of total variation across studies that is due to heterogeneity rather than chance. Publication bias was assessed by the rank correlation test and also confirmed by funnel plot analysis. The reported values were two-tailed, and the hypothesis testing results were considered statistically significant at *p* = 0.05. Statistical analysis was performed using the Comprehensive Meta-Analysis (CMA) software version 3.0 (Biostat, Englewood, NJ, USA).

## 3. Results

Study characteristics: In total, 15 clinical trials in the different phases (2 studies as first-in-man, 3 in phase I, 3 in phase I/II, and 7 in phase II), consisting of 452 cases and 629 controls, were included in our analysis. Regarding the GBM population included in the studies, all studies included only the cases with newly diagnosed GBM.

**Figure 2:**
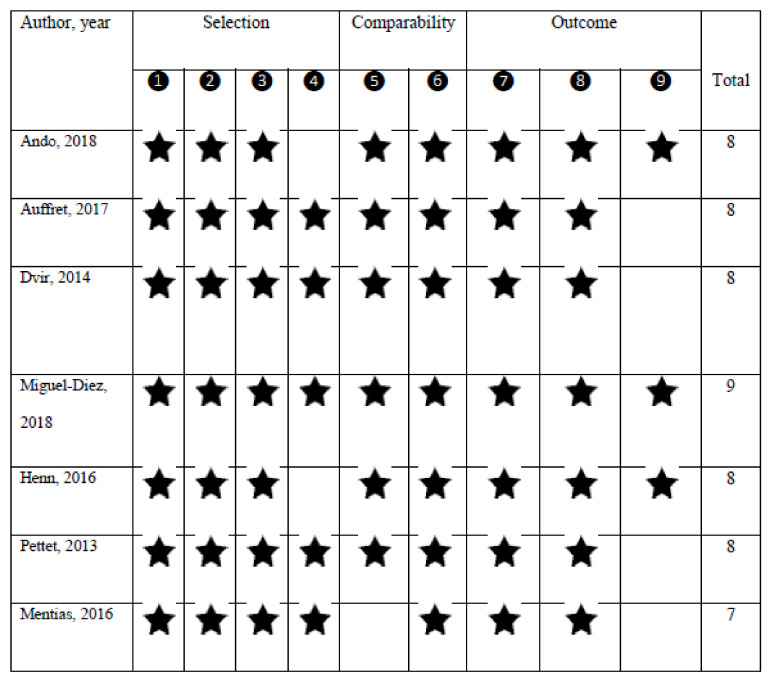
The quality assessment of the studies according to the nine-star Newcastle–Ottawa Scale (NOS) scoring system.

The quality assessment showed a NOS score of 7 or higher for all studies, indicating the presence of high methodological quality (Figure 2).

**Figure 3:**
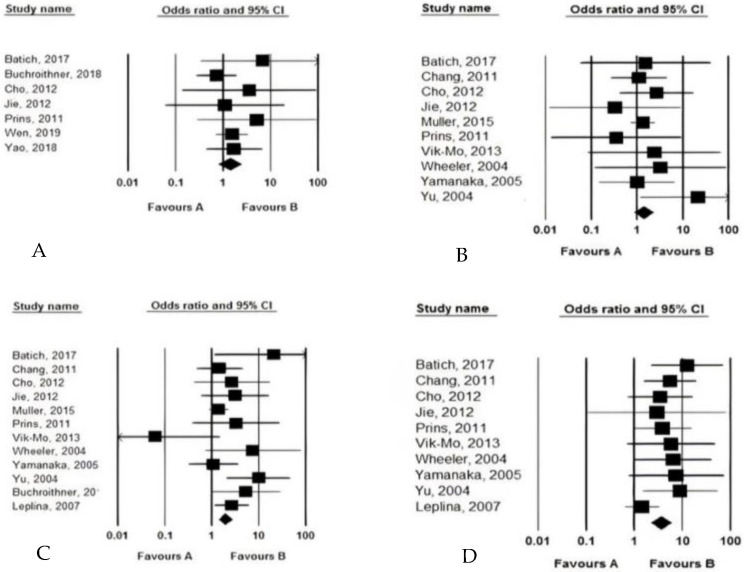
The pooled analysis of the efficacy of dendritic cell vaccination on mid-term survival (**A**: 6-month PFS, **B**: 6-month OS, **C**: 12-month OS, **D**: 24-month OS).

Efficacy outcomes: Among the 15 studies, 15 assessed the overall survival (OS) and progression-free survival (PFS), 12 determined the median OS time (months), 4 assessed the median PFS time (month), 6 assessed the mid-term PFS, and 12 assessed the mid-term OS. The OS and the PFS were significantly different between patients who received the DCV and those who did not. In this regard, using the DCV led to significantly longer OS (weighted mean differences of 5.775, 95% CI: 3.901–7.649, *p* < 0.001), and also longer PFS (weighted mean differences of 1.598, 95% CI: 1.204–1.933, *p* < 0.014). DCV could lead to increased OS and PFS by 5.7 and 1.5 times, respectively. The heterogeneity across the studies in OS and PFS measurements was significantly relevant, with I^2^ values of 91.564 to 92.325, respectively. In terms of comparing the mid-term survival of patients receiving therapeutic regimens with and without considering DCV, we observed no difference between the two groups 6-month PFS (HR = 1.385, 95% CI: 0.822–2.335, *p* = 0.673) and also 6-month OS (HR = 1.408, 95% CI: 0.882–2.248, *p* = 0.754); however, DCV led to significantly longer 1-year OS (HR = 1.936, 95% CI: 1.396–2.85, *p* = 0.001) and longer 2-year OS (HR = 3.670, 95% CI: 2.291–5.879, *p* = 0.001). Hence, introducing the DCV could lead to increased 1- and 2-year survival of patients by 1.9 and 3.6 times, respectively (Figure 3).

Safety outcomes: No side effects were reported following DCV protocols, and drug-related complications were tolerable and reversible (Table 2).

## 4. Discussion

It can be inferred from many animal and human studies that the immune system can help shape the future of cancer treatment by recognizing malignant cells and destroying them efficiently. In fact, this tumor suppression role is mediated by both the cellular and humoral antitumor immune response, especially by CD8^+^ cytotoxic T lymphocytes [37]. Pathologically, T cells in cancer patients have been revealed to exhibit reactivity against tumor biochemical particles, including peptides and proteins derived from the tumor tissue that are sourced by occurring mutations on embryonic genes related to tumor growth and differentiation [38,39]. These potentials can provide new insights into developing therapeutic agents, such as creating vaccines for inhibiting cancer progression and improving patients’ survival. Immunotherapy for GBM comprises various methods, including peptide and dendritic cell vaccines, checkpoint inhibitors, chimeric antigen receptor (CAR) T-cells, and oncolytic virotherapy [40]. Initially, vaccines originated from autologous tumor cells, tumor antigen peptides, or cell lysates generating promising immune responses [41]. However, by applying such methods, no specific immune antitumor response was achieved. In recent decades, one of the major discoveries in tumor immunotherapy has been to prove the critical role of specialized antigen-presenting cells, like the DC in the creation of cell-mediated immune responses by the production of cancer-specific vaccines [42]. The DCV against GBM has garnered special attention. Extensive clinical trials have been designed and conducted to prove its effectiveness and safety, but some have been met with conflicting results. In the present study, we aimed to summarize and interpret the results of these studies in order to reach a credible consensus. Hence, we systematically reviewed 15 clinical trials assessing the DCV efficacy on GBM patients’ survival to ultimately reveal its significant efficacy in improving mid-term OS and OFS in patients with GBM. In other words, the introduction of DCV could effectively prolong patients’ survival and, therefore, inhibit the tumor progression/recurrence. However, two important points are worth considering. The DCV treatment regimen is apparently incapable of generating a clinical measurable response in the short term, with no significant effect on patients’ survival at 6 months. However, after a longer observation time (more than one year), the DCV shows its inhibitory effect on tumor progression.

In a meta-analysis performed by Vatu et al., the DCV resulted in improvements in OS and PFS (35% and 41%, respectively) and it was superior to viral therapy (four clinical trials on herpes simplex virus thymidine kinase/ganciclovir gene therapy were included) in both outcome measures. However, they did not analyze the results at different follow-up times. While there is only a 40% overlap in the final analyzed studies, our work includes 50% more patients in the case group [43]. Another meta-analysis performed by Artene et al. did not observe a significant improvement in OS and PFS by viral therapy, but they found a statistically significant improvement of OS by DCV in both primary and recurrent high-grade glioma. However, despite a trend toward an improvement in PFS, it did not reach a significant threshold. They analyzed 8 studies, including 104 patients in the experimental arm [44]. Cao et al. also found a significantly better outcome in terms of both OS and PFS after antigen-pulsed DC treatment at 1, 1.5, 2, 3, and 4-year time points. Their study included nine clinical trials, six of which are also included in our study [45].

It can be hypothesized that the DCV would require more time to be effective against GBM, so that a more articulated immune response, including a combination of cellular and humoral immunity, could be established. Interestingly, Rangel-Reyes et al. have shown this delay in a mathematical model for dendritic cell treatment. They evaluated common obstacles, such as immunosuppression and poor transfer to lymph nodes, that reduce the effect of the DCV and entered them into a mathematical model, and showed that time can be considered in the model as the gestation time or transport delay of the DCV [46]. In addition, the DCV may have less effect on more invasive glioblastomas. Thus, its effect cannot be detected in a short time, during which these patients could die. In this particular setting, the potential to activate an immune response combining cell therapy with additional immuno-oncology tools, like checkpoint inhibitors (CPI), may generate a faster immune response. There are several clinical trials, such as NCT04201873 (using pembrolizumab) and NCT03014804 (using nivolumab), designed to investigate the efficacy of combined treatment with DCV and CPI. Moreover, one can find case presentations that report a good outcome for such a combination therapy [47]. However, more studies are needed to prove its safety and efficacy.

There are some limitations to the current study. The small number of clinical studies and patients enrolled in the meta-analysis, evaluation of newly diagnosed respectable GBM, differences in patients’ characteristics between these studies, differences in DCV preparation protocols, and variations in concomitant administered therapeutics, may have affected the final analysis. It should be noted that, as shown in Table 1, many studies integrated into this meta-analysis are of a non-randomized or historical type. This may reduce the statistical significance of the analysis.

## 5. Conclusions

Among the different modalities for immunotherapy in glioblastoma, dendritic cell vaccination has gathered considerable attention after some encouraging reports that have shown acceptable levels of efficacy and safety. In the present review, we found that the effect of the DCV needs a minimum 6-month period to become significant—a finding that can be explained by mathematical models and pathophysiology. This vital outcome may explain some of the conflict between different clinical trials. We suggest a revision in the design of future clinical trials to include patients with longer expected survival periods, and also consider the incorporation of combination immunotherapies to boost the effect of the DCV. Nevertheless, the limitations of our work should be taken into account, including the limited number of studies and the fact that it may not be generalizable to recurrent glioblastoma and other high-grade gliomas.

## Figures and Tables

**Table 1:** Original data extracted from included studies.

Author, Year	Trial Phase	Type of Tumor	Sample Size	Mean Age	Male Gender	Follow-Up (Months)	DCV Regimen	Control Group
Batich, 2017 [21]	I	ND ^a^	Case: 11 Control (historical): 23	55 55	8 16	60	pp65 lysosome-associated membrane glycoprotein mRNA-pulsed DCs	60
Buchroithner, 2013 [23]	II	ND	Case: 19 Control (randomized): 21	N/A	N/A	18	Not specified	18
Buchroithner, 2018 [22]	II	ND	Case: 34 Control (randomized): 42	54.6 54.0	29 22	12	Tumor lysate-charged autologous DCs (Audencel)	12
Chang, 2011 [24]	I/II	ND	Case: 17 Control (historical): 63	45 42	N/A	60	Phagocytic DCs co-cultured with autologous glioma cells treated by IFN-gamma and heat-shock treatment and then irradiated with 100 Gy	60
Cho, 2012 [25]	II	ND	Case: 18 Control (randomized): 16	52.1 55.8	8 8	14–56 17–53	Whole-tumor lysate pulsed DCs	14
Jie, 2012 [26]	II	ND	Case: 13 Control (randomized): 12	40.2 43.1	10 9	24	Autologous glioblastoma-DCs (GBM apoptosis induced by heat-shock)	22
Leplina, 2007 [27]	Pilot	ND	Case: 39 Control (historical): 80	43 46	–	36	Interferon-induced DCs	36
Muller, 2015 [28]	II	ND	Case: 117 Control (historical): 165	51.0 52.2	–	36	Not specified	30
Prins, 2011 [29]	I	ND	Case: 23 Control (historical): 68	53 55	16 48	60	Glioma lysate-pulsed DCs booster vaccinations with either imiquimod or poly-ICLC adjuvant	58
Vik-Mo, 2013 [30]	Pilot	ND	Case: 7 Control (historical): 10	57 62	–	24	Dendritic cell-based vaccine targeting cancer stem cells	24
Wheeler, 2004 [32]	I/II	ND	Case: 25 Control (randomized): 13	54 56	16 4	48	Autologous DCs loaded with HLA-eluted peptides from cultured tumor cells or autologous tumor freeze-thaw lysate	48
Wen, 2019 [31]	II	ND	Case: 75 Control (randomized): 42	57.4, 57.5	44, 31	40	DCs pulsed with six synthetic peptide epitopes targeting GBM tumor/stem cell-associated antigens MAGE-1, HER-2, AIM-2, TRP-2, gp100, and IL13Ra2	39
Yamanaka, 2005 [36]	I/II	ND	Case: 18 Control (historical): 27	50 56	–	48	Peripheral blood DCs pulsed with autologous tumor lysate	48
Yao, 2018 [34]	II	ND	Case: 22 Control (Randomized): 21	48, 50	13, 11	14	DCs pulsed with glioblastoma stem cell lysates	12
Yu, 2004 [35]	I	ND	Case: 14 Control (historical): 26	46 53	10 18	60	Autologous DCs pulsed with autologous tumor lysate	60

a: Not defined.

**Table 2:** The outcome of the dendritic cell vaccination strategy.

Author, Year	Number	Median OS	Median PFS	6-Month PFS	6-Month OS	12-Month OS	24-Month OS	Toxicity
Batich, 2017 [21]	Case: 11 Control: 23	41.1 19.2	25.3 8.0	100 78.3	100 95.7	100 52.2	72.7 17.4	No adverse events
Buchroithner, 2013 [23]	Case: 19 Control: 21	14.6 12.7				89.0 62.0		No adverse events
Buchroithner, 2018 [22]	Case: 34 Control: 42	18.8 18.9		66.7 71.4				- Thrombopenia (*n* = 7) - lymphopenia (*n* = 1) - leucopenia (*n* = 2) - rash (*n* = 2) - fatigue (*n* = 3) - headache (*n* = 2) - nausea (*n* = 1)
Chang, 2011 [24]	Case: 17 Control: 63	17.3 12.7			85.1 81.0	64.7 55.6	41.2 11.1	- Lymphopenia (*n* = 17) - serum AST/ALT elevations (*n* = 8) - seizures (*n*= 3) - hydrocephalus (*n* = 1)
Cho, 2012 [25]	Case: 18 Control: 16	ND: 31.9 ND: 15.0	ND: 8.5 ND: 8.0	100 100	88.9 75.0	ND: 88.9 ND: 75.0	ND: 44.4 ND: 18.8	- abnormal liver function (*n* = 1) - mild lymphopenia (*n* = 1)
Jie, 2012 [26]	Case: 13 Control: 12	ND: 17.0 ND: 10.5		ND: 92.3 ND: 91.7	92.3 100	ND: 69.2 ND: 41.7	ND: 7.7 ND: 0.0	- fever (*n* = 2) - red papules (*n* = 1)
Leplina, 2007 [27]	Case: 39 Control: 80					74.4 52.5	35.9 27.5	No adverse events
Muller, 2015 [28]	Case: 117 Control: 165				81.3 76.3	52.3 43.6		No adverse events
Prins, 2011 [29]	Case: 9 Control: 82	31.4 15.9		100 80	100 100	88.9 70.7	55.6 24.4	No adverse events
Vik-Mo, 2013 [30]	Case: 7 Control: 10				100 100	58.7 80.0	71.4 30.0	- Fatigue (*n* = 7) - anorexia (*n* = 5) - focal epileptic seizures (*n* = 1)
Wheeler, 2004 [32]	Case: 13 Control: 13				100 100	92.3 61.5	53.8 15.4	No adverse events
Wen, 2019 [31]	Case: 75 Control: 42	MD: 17 MD: 15	MD: 11.2 MD: 9.0	69.1 60.4				- Nervous system disorder (*n* = 4) - fatigue (*n* = 3) - musculoskeletal disorder (*n* = 1) - blood disorders (*n* = 6) - infections (2) - metabolic disorders (*n* = 9) - skin disorders (*n* = 8)
Yamanaka, 2005 [36]	Case: 18 Control: 27				88.6 88.6	61.1 59.3	22.2 3.7	No adverse events
Yao, 2018 [34]	Case: 22 Control: 21	MD: 17.3 MD: 10.7		77.2 66.7				- fever (*n* = 1) - erythema (*n* = 1)
Yu, 2004 [35]	Case: 14 Control: 26	33.2 7.5			100 57.7	78.6 26.9	42.9 7.7	No adverse events

Publication bias: The heterogeneity across the studies in assessing the efficacy of DCV on mid-term survival was insignificant, with I^2^ values ranging from 0.0 to 0.39, and Egger test excluded non-significant publication bias in the analyses.
